# Utilization of Secondary Jet in Cavitation Peening and Cavitation Abrasive Jet Polishing

**DOI:** 10.3390/mi13010086

**Published:** 2022-01-05

**Authors:** Hao Pang, Gracious Ngaile

**Affiliations:** Department of Mechanical and Aerospace Engineering, North Carolina State University, 1840 Entrepreneur Dr., Campus Box 7910, Raleigh, NC 27695, USA; hpang2@ncsu.edu

**Keywords:** cavitation, peening, jet polishing

## Abstract

The cavitation peening (CP) and cavitation abrasive jet polishing (CAJP) processes employ a cavitating jet to harden the surface or remove surface irregularities. However, a zero incidence angle between the jet and the surface limits the efficiency of these two processes. This limitation can be improved by introducing a secondary jet. The secondary jet interacts with the main jet, carrying bubbles to the proximity of the workpiece surface and aligning the disordered bubble collapse events. Through characterizing the treated surface of AL6061 in terms of the hardness distribution and surface roughness, it was found out that the secondary jet can increase the hardening intensity by 10%, whereas the material removal rate within a localized region increased by 66%. In addition, employing multiple secondary jets can create a patched pattern of hardness distribution. Another finding is that the hardening effect of the cavitation increases with the processing time at first and is then saturated.

## 1. Introduction

Most failures in engineering materials, such as fatigue fracture, fretting, wear, and corrosion, originate from the exterior layer and are sensitive to the structure and properties of the material surface. The fatigue strength of a product can be enhanced by producing compressive residual stress, increasing the surface hardness, and through grain refinement. To increase fatigue life, many components in the aerospace and automotive sectors undergo shot peening, in which the surface is bombarded with hard balls to induce compressive residual stresses [1,2,3,4]. However, this method results in excessive surface roughness, and the balls might fail to reach intricate areas of the part. It should be noted that substantial surface roughening can result in unexpected failures in low-cycle fatigue loading situations [5,6]. In recent years, cavitation peening (CP) processes have attracted much attention due to their advantages over the conventional surface treatment processes, such as shot peening and laser peening [7,8,9,10].

**Figure 1:**
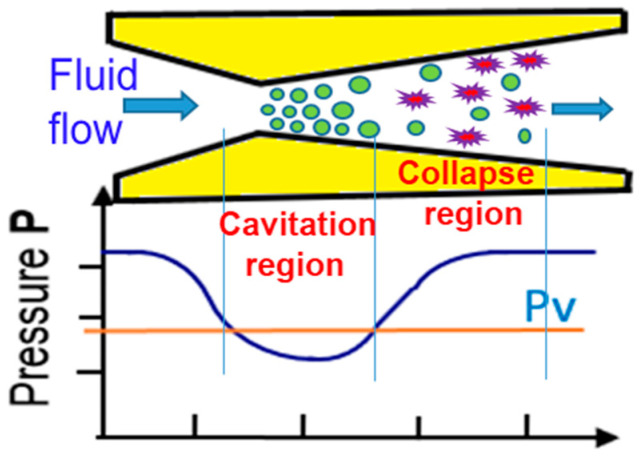
Cavitation in a Venturi tube.

In the cavitation peening (CP) process, cavitation is formed as a fluid passes through a Venturi-type nozzle or an orifice. Upon impact with the workpiece, the imploded bubbles supply energy for the peening process. Figure 1 shows the formation of cavitation bubbles in a Venturi-type nozzle. The figure also shows the pressure trajectory along the fluid flow path and vapor pressure, Pv. The throat geometry of the nozzle creates a pressure drop and leads to vaporization of the liquid, which is referred to as cavitation. These vaporous bubbles exiting the nozzle bear tremendous energy, which is released in the form of the pressure waves and micro-jets when they collapse under a high recovery pressure. The nozzle can be designed such that intense bubble collapse occurs at the surface of the workpiece. The impact of the pressure waves and the micro-jets on the workpiece surface results in local plastic deformation and work hardening of the workpiece material [11,12]. This action also induces compressive stresses on the surface of the workpiece.

Cavitation peening has been found to be superior in enhancing surface hardness and fatigue strength, compared to other traditional methods. In an extensive experimental study, Soyama et al. [11] compared the fatigue strengths of stainless-steel specimens treated using the cavitation peening, water jet peening, laser peening, and shot peening techniques. Cavitation peening performed the best, followed by shot peening, laser peening, and finally water jet peening. The same conclusions were drawn by Takahashi et al. [13] and Sonde et al. [14], who studied the performance of the cavitation peening and shot peening processes with regards to enhancing surface hardness, surface finish, fatigue strength, and corrosion resistance. Water cavitation peening experiments on Al 7075-T651 conducted by Marcon et al. [15] yielded compressive residual stresses as high as 400 MPa and as deep as 350 µm below the surface. This shows the effectiveness of cavitation peening compared to shot peening.

**Figure 2:**
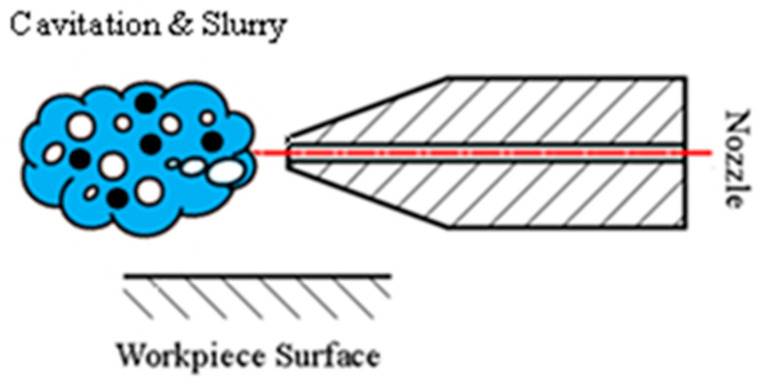
Cavitation abrasive jet polishing.

Similarly, in cavitation abrasive jet polishing (CAJP), the slurry, which is a mixture of abrasive particles and fluid, is pumped through a nozzle to generate cavitating jets, which are ultimately used to polish the surface of the workpiece. Figure 2 shows a schematic diagram of CAJP. The abrasive particles with high kinetic energy flatten or cut off irregular asperities on the workpiece surface, leading to lower surface roughness. In CAJP, the energy emanating from collapsing bubbles is also used to remove the asperities and accelerate abrasive particles via the pressure waves and the micro-jet, thus enhancing polishing efficiency. Nagalingam et al. [16] employed the CAJP to improve the roughness of internal surfaces. In that study, a 47.5% reduction in surface roughness was achieved in three hours. Precision polishing of hard, brittle materials such as silicon, which is widely used for optical components, is challenging. Regardless of the particle size of abrasives, traditional contact grinding and polishing methods are bound to produce microcracks on the surface [17]. Zhao et al. [18] developed a polishing system with multiple Venturi cavitation channels to form a rotary abrasive flow above the workpiece. Polishing for 8 hours yielded a surface roughness of 7.8 nm, as compared to 10.5 nm in a non-cavitation-polished workpiece.

Compared to their conventional counterparts, CP and CAJP are more suitable for treating surfaces with complicated geometries. They can also be used to process internal surfaces since they employ fluid as the work medium. The heat damage to the workpiece is minimal since the fluid continuously removes the heat during the process.

**Figure 3:**
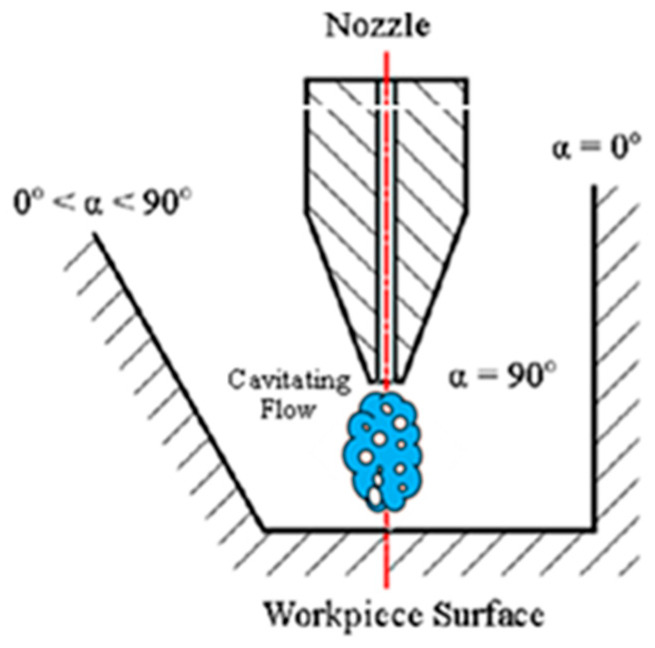
Cavitation peening (CP).

The previous research has demonstrated the positive role of cavitation in peening and jet polishing. However, these two processes are confronted with several challenges. First, when CP is used to treat workpieces with complicated geometries, the incidence angle α, shown in Figure 3, may be zero. Qin et al. [19] found out that the efficiency of the process is sharply decreased with a decrease in the incident angle. Changing the incidence angle by incorporating a mechanism to rotate the nozzle or the workpiece may be limited by the spatial interference between them. Second, when the incidence angle α approaches zero, quantification of the hardness distribution created by the CP, which is critical to the quality of the product, becomes uncertain. Third, there is the potential for further improvements of the efficiency of CAJP, particularly when the cavitation energy is not fully harnessed. For example, bubbles which collapse far away from the workpiece surface and micro-jets which are not directed towards the surface are underutilized [20,21].

Cases in which the incidence angle is zero or the workpiece surface is parallel to the cavitating jet are commonly seen in CP and CAJP, especially in the latter. In this study, a cavitation device which mimics the case of a zero incidence angle or a parallel type of workpiece-nozzle configuration was developed. A novel approach was proposed to control the hardness distribution and enhance process efficiency by introducing secondary jets. The hardness distribution and surface roughness of the treated specimens using this approach were measured and analyzed.

## 2. Multiple-Jet Cavitation Approach

**Figure 4:**
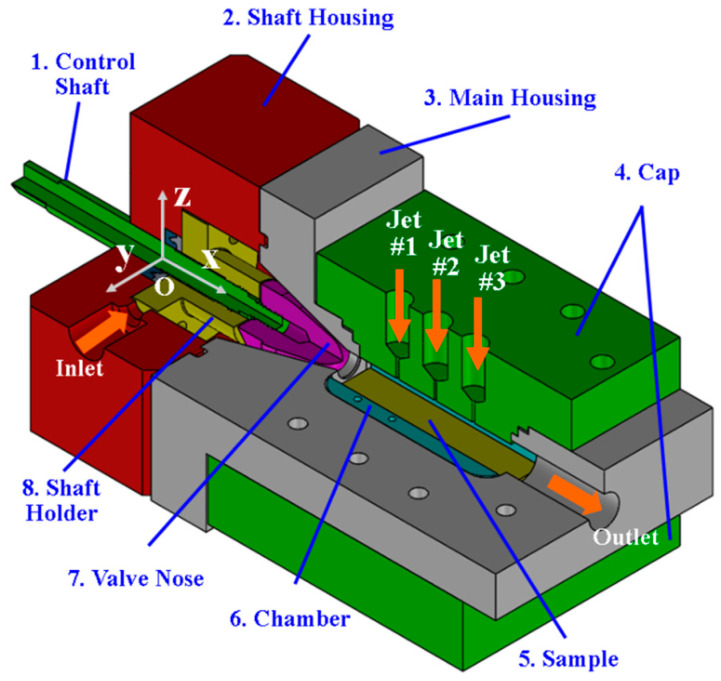
Multiple-jet cavitation device.

**Figure 5:**
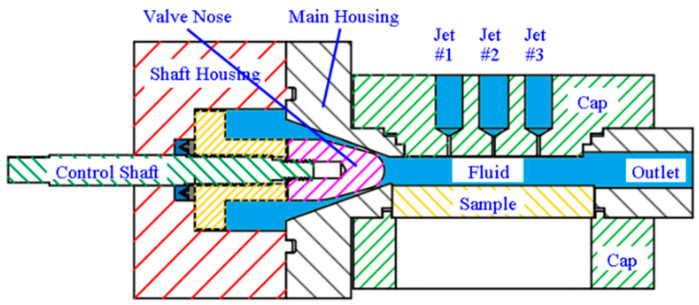
Cutaway of the cavitation device.

In order to address the situation of a zero incidence angle, a cavitation device with a multiple-jet configuration, as shown in Figure 4, was developed. Figure 5 shows a cutaway of the device on the *x-z* plane. The fluid is pumped through the nozzle formed by a valve nose (#7) and the conical wall of the main housing (#3) and then enters the chamber (#6). When the jet exists through the nozzle, a shear layer is formed between the high-speed jet and the relatively static fluid in the chamber (#6). The large velocity gradient in this shear layer gives rise to a vortex, which further forms a low-pressure zone at its center. This low-pressure zone is the birthplace of the cavitating bubbles. In addition, the turbulence of the jet also encourages the inception of cavitation. The inception of cavitation and cavitation intensity are usually judged using the so-called cavitation number, which can be approximated as the ratio of the downstream pressure in the chamber (#6) to the pressure drop across the nozzle. The lower the cavitation number is, the more likely cavitation is to occur and the bubble cloud shed from the jet is more vaporous. The pressure drop ΔP across the nozzle, which is defined as the pressure in the upstream of the constriction minus its counterpart in the downstream, can be changed by advancing or retracting the valve nose (#7) via a control shaft (#1). The control shaft (#1) can translate the valve nose (#7) by 3–4 mm so that the minimum gap of the constriction varies from 0.3 mm to 0.8 mm. The gap in this range leads to a pressure drop from 4 MPa to 6 MPa, which corresponds to cavitation numbers ranging from 0.016 to 0.024. The small end of the valve nose has a diameter of 12 mm and its semi-taper angle is 15°.

**Figure 6:**
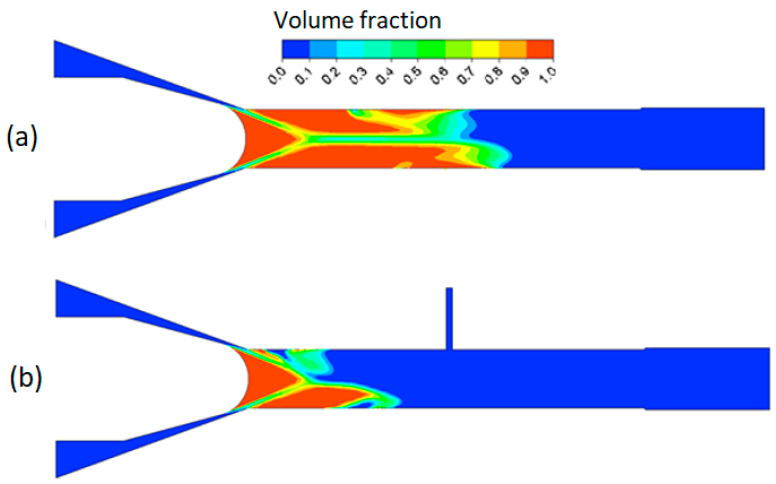
Vapor volume fraction (**a**) without secondary jet and (**b**) with secondary jet.

**Figure 7:**
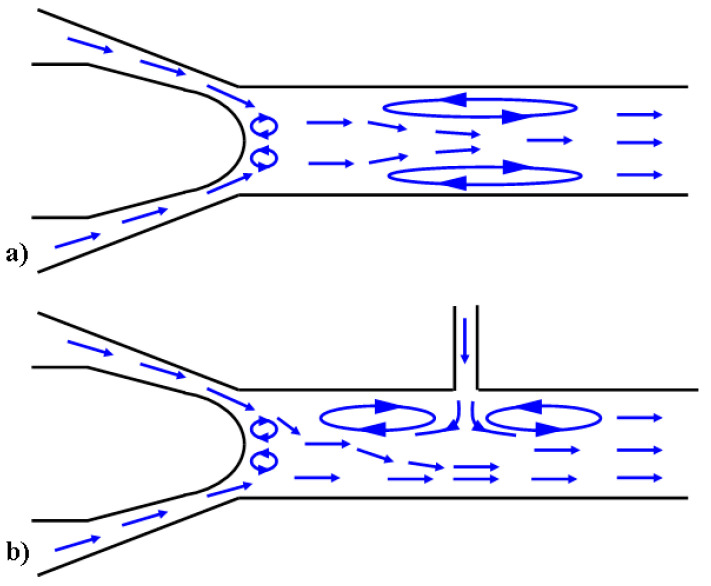
Velocity field (**a**) without secondary jet and (**b**) with secondary jet.

The bubbles exiting from the valve nose flow through the chamber (#6), where a specimen for surface treatment is attached (#5). The proposed approach employs secondary jets (Jet #1, #2, and #3) as seen in Figure 4 and Figure 5. The secondary jet is ejected from the slender hole with a bore diameter of 1 mm and a length of 6 mm. These secondary jets are not cavitating and they cross the cavitation jet from the main nozzle. Figure 6a,b show that the intersection of the cavitating flow from the main nozzle and the secondary jet creates a high-pressure zone. This pressure gradient causes the bubbles to drift towards the specimen surface, where they will collapse. Figure 6a,b show vapor-volume fraction distributions obtained from CFD simulations, where a volume fraction of one indicates water vapor and volume fraction of zero indicates liquid water. Utilization of the secondary jet has the potential to enhance peening intensity and increase material removal rates, and provides the opportunity to control the hardening pattern on the workpiece. The secondary jet brings about two major benefits. First, the secondary jet carries the bubbles to the proximity of the workpiece surface. As shown in Figure 7a, in the absence of the secondary jet, two flat vortexes may develop in the chamber and are attached to the chamber wall and specimen surface, respectively. The vortex in adherence to the specimen surface deflects many bubbles away from the specimen. In contrast, Figure 7b shows that the secondary jet may prevent the formation of the vortex on the specimen surface and forces all bubbles to approach the specimen and collapse above it. This results in less attenuation of the water hammer pressure and the pressure wave amplitude. The water hammer pressure from the microjets and the pressure wave emanating from several rounds of bubble collapse are two main mechanisms that facilitate surface hardening or the removal of material from the surface. The formulas for microjet velocity and the water hammer pressure were presented in Brennen’s and Kodama’s works [22,23]. Equation (1) shows that the microjet velocity $U_{jet}$ is proportional to the coefficient, γ. The coefficient γ is a dimensionless number, which is a function of the bubble size and the distance, *L*, from the collapse event to the solid surface. The coefficient γ decays with increasing L and it has been reported to take a value of 7.6 [22]. By combining Equations (1) and (2), it can be seen that the water hammer pressure $\left(P_{wh}\right)$ diminishes as the bubble-to-surface distance increases. In addition to the water hammer pressure, Reisman et al. [24] pointed out that the pressure wave $\left(P_{sw}\right)$ is inversely proportional to the distance L, which is shown in Equation (3). Note: Figure 7 is a schematic diagram of the velocity field, which is derived from the CFD simulation.

(1) $$U_{jet}=\gamma \sqrt{\frac{P_{am}-P_{V}}{\rho_{L}}}$$

(2) $$P_{wh}=c\rho_{L}U_{jet}$$

(3) $$P_{sw}=\frac{\rho_{L}}{4\pi L}\frac{d^{2}V}{dt^{2}}$$

**Figure 8:**
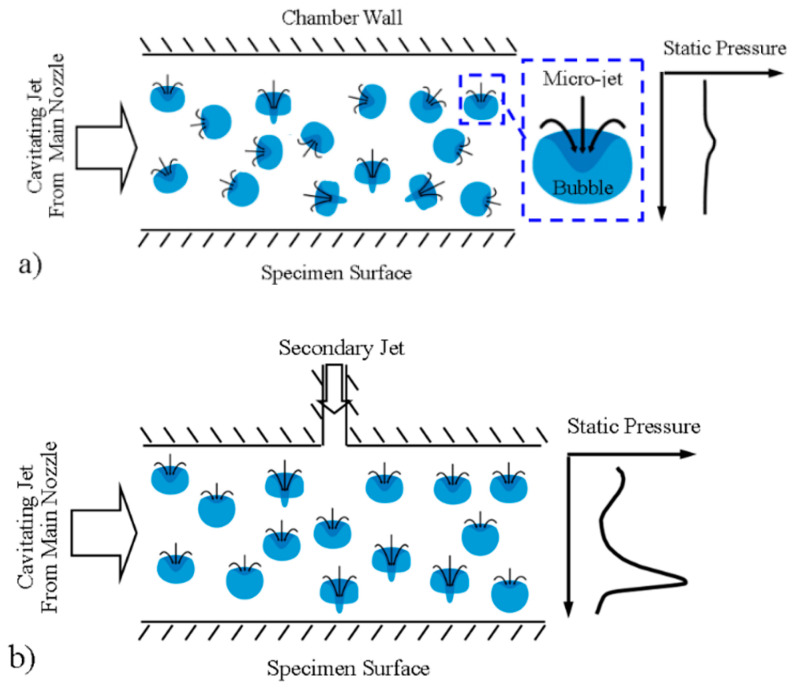
Illustration of cavitation (**a**) without and (**b**) with potential realignment using a secondary jet.

Second, the secondary jet creates a pressure gradient, resulting in asymmetric bubble collapse. The asymmetry implies that the pressure is higher on the side of the bubbles far from the surface than on the side of the bubbles close to the workpiece surface. Figure 8a shows an illustration of chaotic micro-jets that may be encountered in cavitation flow. Figure 8b shows a scenario in which micro jets can be realigned towards the solid surface in the presence of a secondary jet. The secondary jets also provide design opportunities for shortening of the distance from the bubble collapse sites to the workpiece surface. As shown in Figure 8a, the static pressure in the spanwise direction, which is the *z* axis in Figure 4, is almost unvarying, which is equivalent to a zero pressure gradient. In contrast, after the introduction of the secondary jet, a surge in the pressure appears in the flow right above the specimen, which creates a significant asymmetry for the bubble collapse event. Note: the static pressure in Figure 8 is obtained from the CFD simulation and it is normalized by a reference value, which is the minimum pressure of the pressure profile.

## 3. Test Setup

**Figure 9:**
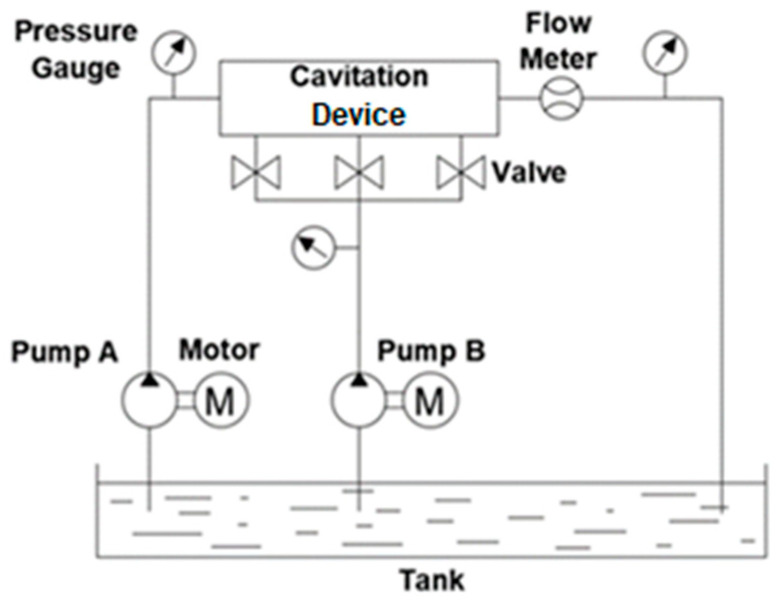
Hydraulic circuit of the overall system.

**Figure 10:**
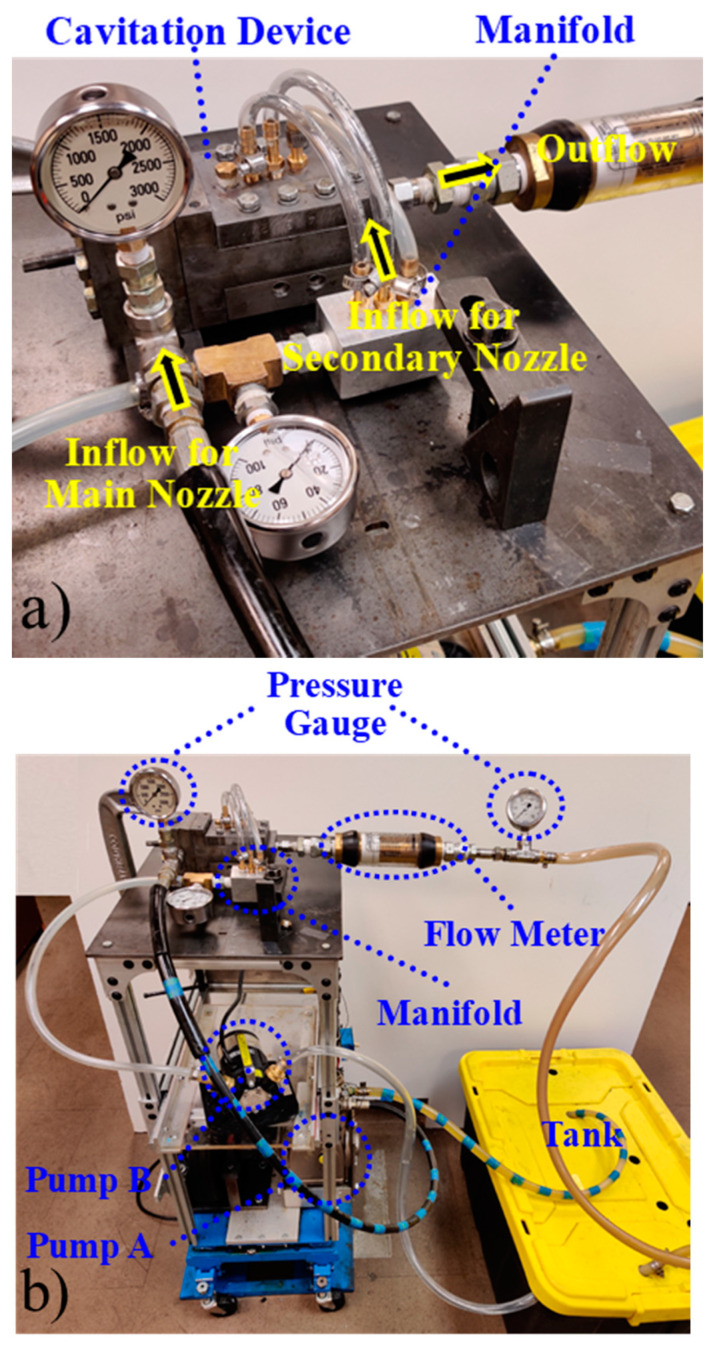
Experimental setup (**a**) Enlarged top view of the setup, (**b**) Overall setup.

The cavitation device (Figure 4) is connected to the system as shown in Figure 9. Pump A sucks the water from the tank and pushes it through the cavitation device. Pump A is a plunger-type pump, which operates at a speed of up to 1750 RPM and discharges the fluid at the maximum flow rate of 18 L/min. A 2.25 kW motor transmits the torque to pump A via a belt drive. Pump B is used to drive the secondary jet. Because there are three locations for the secondary jets in the cavitation device, a manifold is used to distribute the fluid and each channel can be independently opened and closed. The fluid flows from pump A and pump B interact in the cavitation device shown in Figure 4, among which the fluid flow from pump A is cavitating, whereas the fluid flow from pump B is non-cavitating. Figure 10a shows how these flows interact in the cavitation device. In order to monitor the pressure drop created by the cavitation device, two pressure gauges are used. In addition, another pressure gauge displays the discharge pressure from pump B. Finally, a flow meter is used to measure the total flow rate from pump A and B. The overall setup is shown in Figure 10b.

## 4. Materials and Procedures

The cavitation experiments were performed to treat the surfaces of Aluminum 6061 TO specimens with the dimensions of 63 mm × 25 mm × 9 mm. This material has a Knoop hardness (HK) of 41.1–43.9 kgf/mm^2^. In order to evaluate mass removal by cavitation, all specimens were kept to the same surface roughness before processing. The average surface roughness $R_{a}$ was 0.11–0.14 μm and valley depth surface roughness $R_{v}$ was 0.58–0.59 μm.

At the beginning of the cavitation process, the specimen was put into the chamber (#6) and pump A was turned on. The pressure drop between the inlet and outlet of the cavitation device was set to 4.1 MPa by adjusting the control shaft (#1). The main nozzle outputs the cavitating flow at the flow rate of 14 L per min. Subsequently, pump B was switched on to activate the secondary jet. The inlet pressure of the secondary jet was 138 KPa and its flow rate was 3 L per min. By maneuvering the manifold, different combinations of the secondary jet could be utilized to treat the specimens. For each combination of the secondary jet, three specimens were processed.

The processed specimens were characterized in terms of hardness and surface roughness. The Knoop hardness test was carried using a Leco M400 microhardness tester. In the hardness test, the specimen was placed on the stage of the tester and aligned with the two axes of the stage. In order to acquire the hardness distribution across the surface, the hardness was measured at equally spaced points, each of which had a unique coordinate. At each point, four measurements were taken within a 1.2 mm × 1.2 mm area. The average of these four measurements were recorded, together with the error bar.

The surface roughness was measured using a Mitutoyo SURFTEST SJ-310. This stylus-type detector had a resolution of 0.02 μm. In measuring the surface roughness, ISO 4287-1997 was implemented with a cut-off length, $\lambda_{c}$, of 0.8 mm. The number of sampling lengths used was five. For each specimen, four measurements were taken and averaged.

Micrographs of the surface morphology were obtained using a Keyence VKx1100 confocal laser scanning microscope (CLSM). The magnification factor of the objective lens was 20× and the spatial resolution was 0.13 μm. A high-accuracy-level scanning pitch was employed.

## 5. Results and Discussion

In order to study the effects of the secondary jet on peening or hardening intensity, the hardness distribution on the treated surface was evaluated. The specimens with no secondary jet and with a secondary jet (#2) were compared in terms of the hardness distribution along the centerline of the specimen and were labeled as AL_NJ and AL_J2, respectively. The processing time for AL_NJ and AL_J2 was 10 min. Note: the centerline refers to *x*-axis in Figure 4.

**Figure 11:**
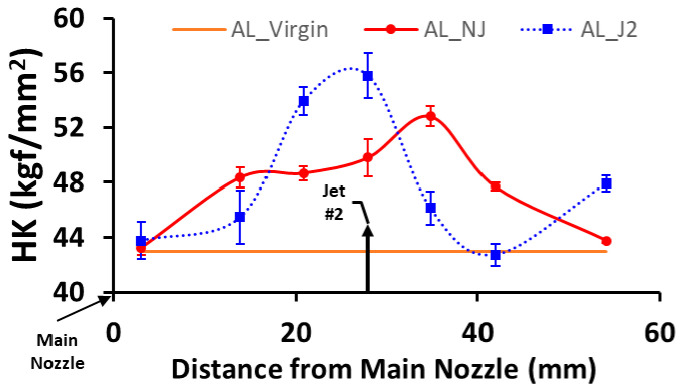
Hardness distribution on the specimen with no jet and with a secondary jet.

We found that the secondary jet affected the hardness pattern and increased the hardening efficiency in a localized area. As shown in Figure 11, in the absence of the secondary jet, the hardness of AL_NJ increased at first with the distance from the nozzle and then decreased. In contrast, when the secondary jet was employed, a localized hardening phenomenon was observed on the specimen region corresponding to the jet location. The rest of the region was slightly hardened. This indicates that the secondary jet can concentrate the release of the cavitation energy within the neighborhood of the jet location and harden a localized region. Meanwhile, the maximum hardness achieved by the secondary jet was 10% higher than the case without the secondary jet. It can be seen that another hardness peak appeared at the outlet end of AL_J2, which indicates that some bubbles could not implode at the secondary jet location. Note: In Figure 11, AL_Virgin refers to the unprocessed specimens, which had HK values of 41.3–43.9 kgf/mm^2^, averaging about 43 kgf/mm^2^.

When the length-averaged hardness is computed from the hardness curve in Figure 11, the average hardness values of the AL_NJ and AL_J2 are 45 kgf/mm^2^ and 45.2 kgf/mm^2^, respectively. The closeness between the two average hardness values for AL_NJ and AL_J2 implies that the secondary jet may only be spatially redistributing the release of cavitation energy but does not alter the total amount of energy. This assertion will hold if the number of collapsed bubbles in the cavitation stream remains constant. However, the results from the hardness tests alone are not sufficient to provide such a conclusion because bubble implosions that occur close to the specimens do not account for all collapsed bubbles in the fluid stream.

**Figure 12:**
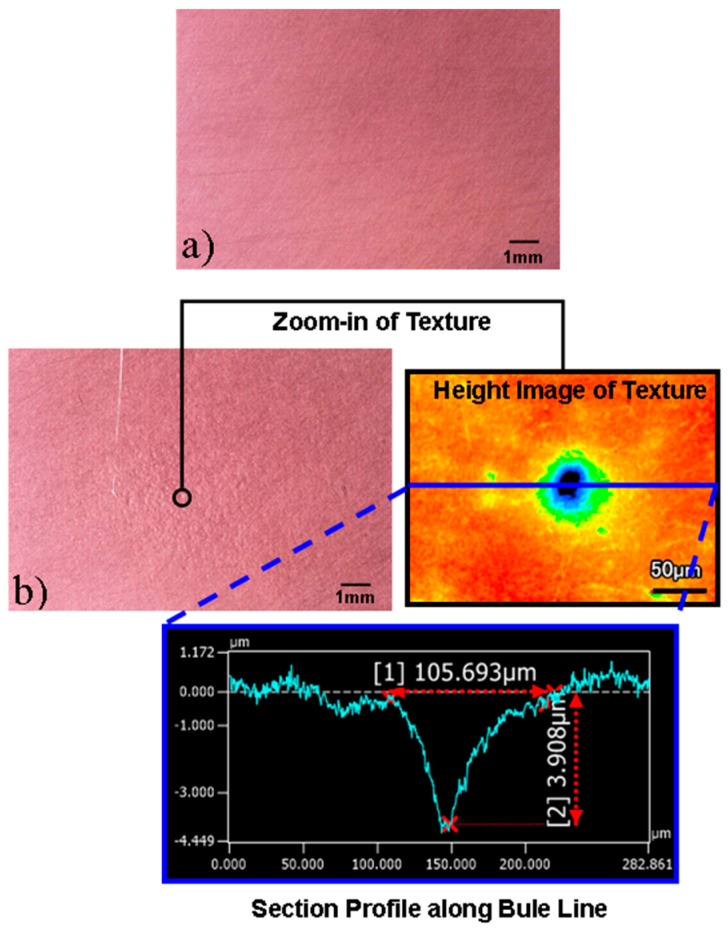
Surface morphology (**a**) before cavitation virgin surface and (**b**) after cavitation.

In addition to strengthening the surface, the cavitation also alters the surface morphology of the workpiece, which is a dominant mechanism of removing surface irregularities in CAJP. The difference in the surface morphology of the specimen before and after cavitation-based treatment is clear when comparing Figure 12a,b. Before cavitation, the virgin material had a polished surface. After cavitation, the specimens exhibited a distinct texture morphology, as seen in Figure 12b. Zooming in further, a cluster of dents can be observed. Figure 12b shows the height and depth dimensions of one of these dents.

**Figure 13:**
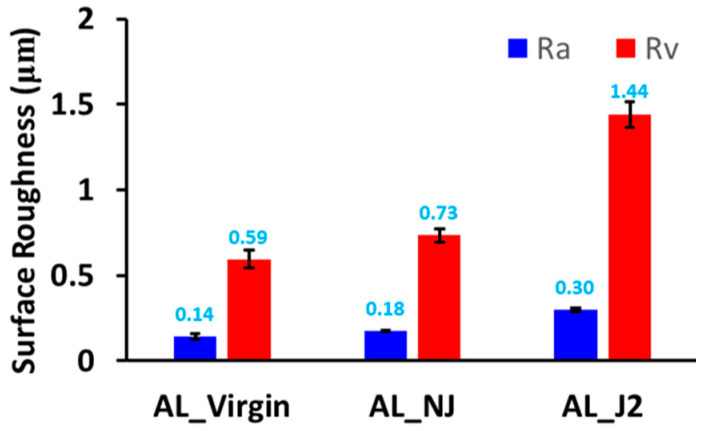
Surface roughness ($R_{a}$ and $R_{v}$ ) of specimens with no jet and with a secondary jet.

Figure 13 shows that in the most hardened region of AL_NJ and AL_J2, the surface roughness was higher in AL_J2 than in AL_NJ. The surface roughness values $R_{a}$ and $R_{v}$ of AL_J2 were 67% and 90% higher than those of AL_NJ, respectively. In addition, the dents that were visible to the naked eye on AL_J2 were not observed on AL_NJ. These findings indicate that the secondary jet can locally increase the material removal rate by concentrating the cavitation energy.

The surface treatment results observed with a single secondary jet (Jet #2) clearly show that the jet can be used to locally enhance hardening intensity and increase material removal rate.

**Figure 14:**
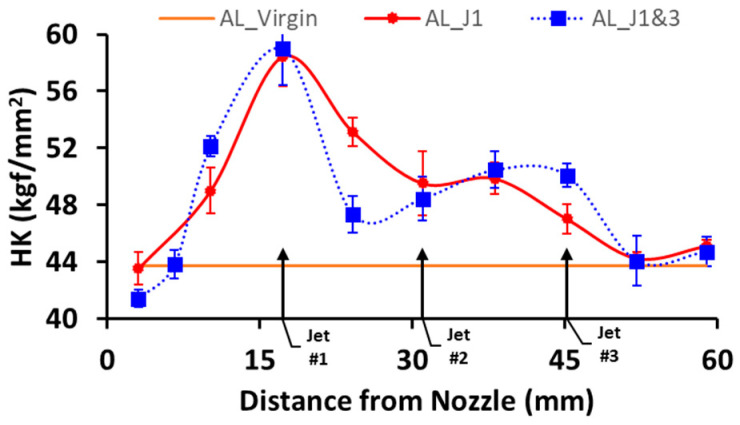
Hardness distribution on the specimen with Jet #1 and with a combination of Jet #1 and Jet #3.

The influence of multiple secondary jets on the hardening pattern was also examined. Jet #1 and Jet #3 were used (Figure 4). Figure 14 shows that a single jet resulted in a hardness profile with a single peak, whereas two secondary jets led to two peaks. Among these peaks, the upstream one was higher than the downstream one. This may be attributed to the following reasons. Bubbles exiting from the main nozzle release most of their energy at the location of the upstream jet. This also implies that there should be a decrease in the density of cavitating bubbles that are about to collapse at the downstream location if a large percentage of these bubbles have already collapsed. Although the introduction of the downstream jet will increase bubble collapse, this will be proportional to the incoming bubbles from the upstream regions.

These observations imply that there is the potential to create a patched pattern of hardening if multiple secondary jets are used.

Since the process efficiency was of interest in this study, the effects of the processing time on the hardness distribution and surface roughness were examined. In order to examine the role that the processing time plays, it was extended up to 40 min and divided into four levels; t1 (10 min), t2 (20 min), t3 (30 min), and t4 (40 min), which corresponded to the specimens labeled as AL_t1, AL_t2, AL_t3, and AL_t4, respectively. For this investigation, secondary Jet #2 was used.

**Figure 15:**
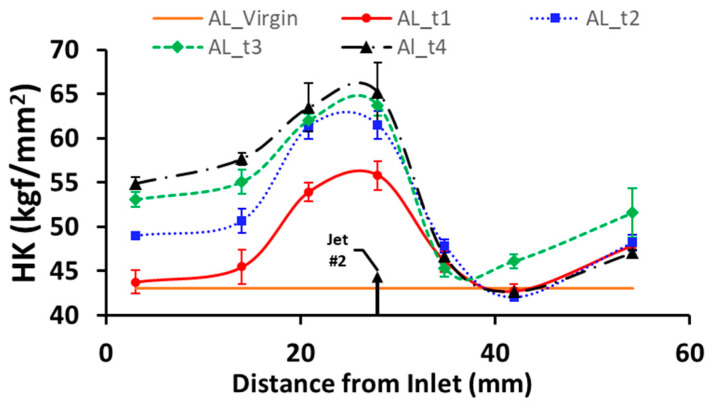
Hardness distribution under various process times.

**Figure 16:**
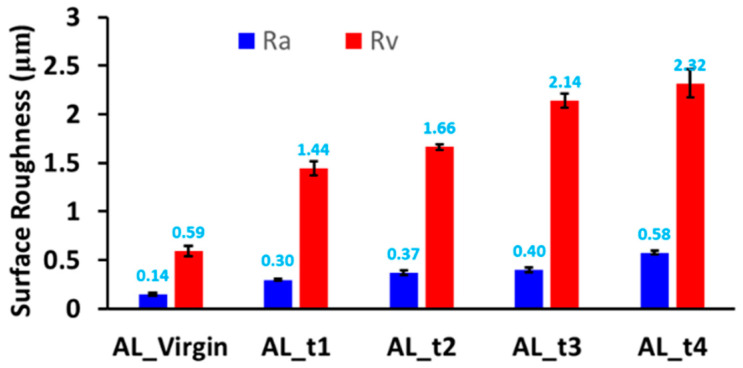
Surface roughness under various process times.

The evolution of the hardness distribution with respect to the processing time is shown in Figure 15. It was found that the maximum hardness near the jet location increased with the processing time at first and was then saturated. The saturation can be explained by the plateau of the flow stress curve of the specimen material (Aluminum 6061 TO) under high strain. After 20 min, a further increase in the processing time yielded no substantial increase in the maximum hardness. A slight expansion of the hardened zone was observed with increase in the process time. The influence of process time on surface roughness is presented in Figure 16, which shows that the surface roughness increases monotonically with an increase in processing time.

## 6. Conclusions

In this study, a novel approach was proposed to improve cavitation peening (CP) and cavitation abrasive jet polishing (CAJP). The premise behind the approach is the introduction of multiple secondary jets, which interact with the main cavitating flow. This approach provides design opportunities for shortening the distance from the bubble collapse sites to the workpiece surface. The secondary jets also align the disordered micro-jets towards the surface. The major conclusions drawn from this study are as follows.

The secondary jet can concentrate the release of cavitation energy within a specific region, increasing the hardening intensity. The maximum hardness achieved by the secondary jet was 10% higher than in the case without a secondary jet.

- Under the same process conditions, the surface roughnesses found in the cases with and without the secondary jet were $R_{v}=0.44\;\mu m$ and $R_{v}=0.73\;\mu m$, respectively. This contrast shows that the secondary jet can enhance material removal rate, which is favorable for the process efficiency of CAJP.
- The hardening effect of the cavitation increases with the processing time at first and is then saturated. However, the surface roughness monotonically increases with the processing time.
- The study demonstrates the potential for creating patched hardening patterns of interest by employing multiple secondary jets.

## Nomenclature

$U_{jet}$	Microjet velocity
γ	Coefficient
$P_{am}$	Ambient pressure
$P_{V}$	Vapor pressure
$\rho_{L}$	Liquid density
c	Sound speed in liquid
$P_{sw}$	Shock wave amplitude
L	Distance from bubble to the solid surface
V	Bubble volume
$P_{wh}$	Water hammer pressure
